# Two-dimensional molecular brush-functionalized porous bilayer composite separators toward ultrastable high-current density lithium metal anodes

**DOI:** 10.1038/s41467-019-09211-z

**Published:** 2019-03-25

**Authors:** Chuanfa Li, Shaohong Liu, Chenguang Shi, Ganghao Liang, Zhitao Lu, Ruowen Fu, Dingcai Wu

**Affiliations:** Materials Science Institute, PCFM Lab and GDHPRC Lab, School of Chemistry, Sun Yat-sen University, Guangzhou, 510275 China

## Abstract

Lithium metal batteries have been considerably limited by the problems of uncontrolled dendritic lithium formation and the highly reactive nature of lithium with electrolytes. Herein, we have developed functional porous bilayer composite separators by simply blade-coating polyacrylamide-grafted graphene oxide molecular brushes onto commercial polypropylene separators. Our functional porous bilayer composite separators integrate the lithiophilic feature of hairy polyacrylamide chains and fast electrolyte diffusion pathways with the excellent mechanical strength of graphene oxide nanosheets and thus enable molecular-level homogeneous and fast lithium ionic flux on the surfaces of electrodes. As a result, dendrite-free uniform lithium deposition with a high Coulombic efficiency (98%) and ultralong-term reversible lithium plating/stripping (over 2600 h) at a high current density (2 mA cm^−2^) are achieved for lithium metal anodes. Remarkably, lithium metal anodes with an unprecedented stability of more than 1900 h cycling at an ultrahigh current density of 20 mA cm^−2^ are demonstrated.

## Abstract (web summary)

The authors use polyacrylamide grafted graphene oxide as a molecular brush to coat the commercial polypropylene separator. The bilayer design combines lithiophilicity chemistry and high mechanical strength, rendering high performance Li metal anodes and offering a strategy for the design of separators.

## Introduction

Research on high-performance electrochemical energy storage has been pursued worldwide to fulfill the needs of emerging high-energy-demand applications, such as portable electronics, electric vehicles, autonomous aircraft, and grid storage^1–4^. Among the myriad electrochemical energy-storage technologies, lithium-ion batteries (LIBs) have monopolized the major markets since their commercialization in 1991 because of their intrinsic high-energy density, light weight, and minimal memory effect^5^. Unfortunately, due to the limited theoretical specific capacities of the widely used commercial anode (LiC_6_) and cathode materials (LiCoO_2_), the highest energy density that traditional LIBs can deliver even when fully developed is far from the demands of these emerging applications^6–9^. With the highest theoretical specific capacity (3860 mAh g^−1^, 10 times that of commercial graphite anodes) and the lowest redox potential (−3.04 V vs. the standard hydrogen electrode) among all anode materials, lithium (Li) metal has long been regarded as the “Holy Grail” anode of Li-based batteries and is a promising candidate for next-generation energy-storage systems with high-energy cathode materials, such as sulfur and oxygen^10–12^.

**Fig. 1:**
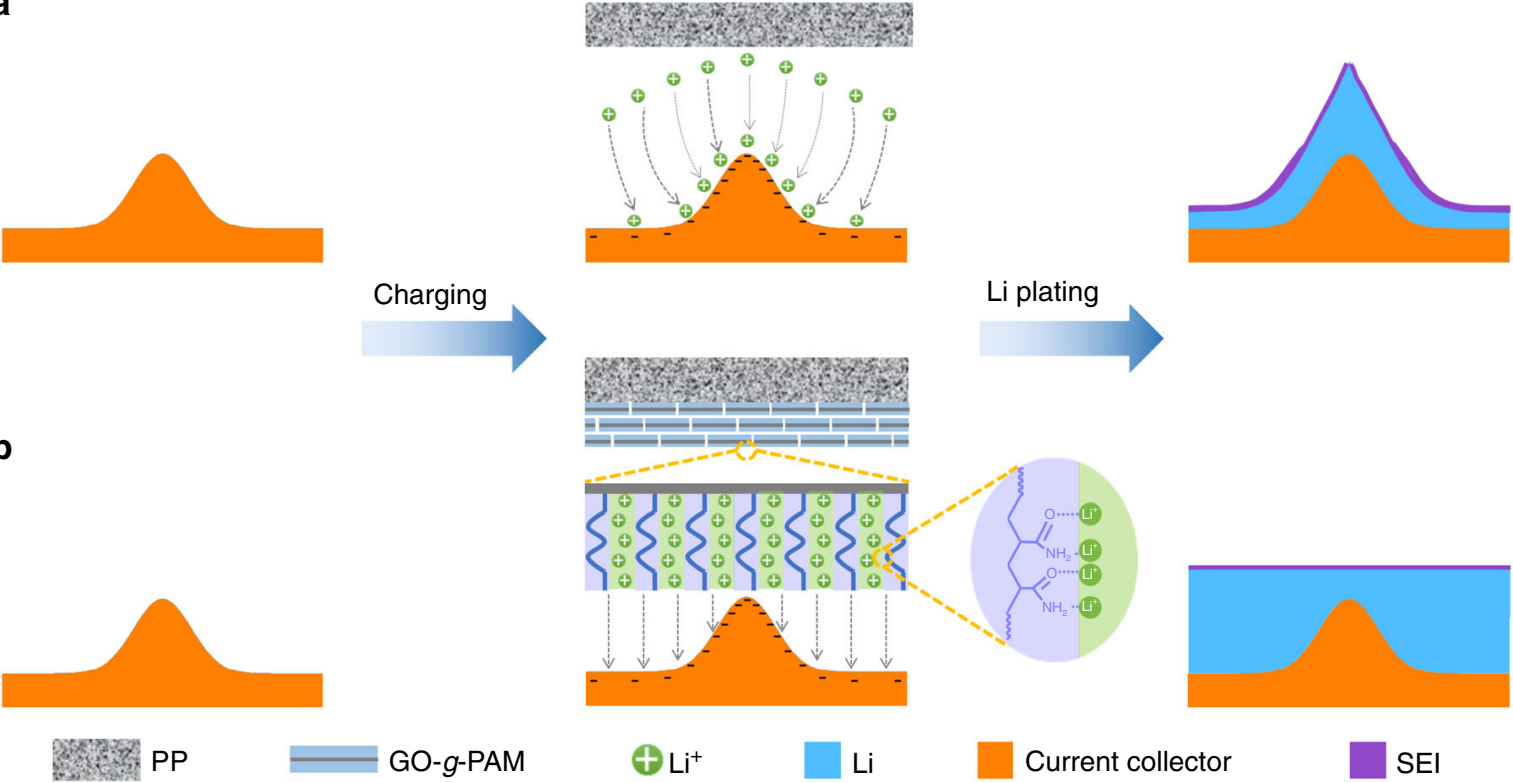
Schematic diagram of Li deposition on electrodes with microscopic surface roughness. **a** Schematics showing Li deposition on an electrode with a PP separator. Because of the stronger electrical field, Li ions will aggregate near the protuberant tip, leading to the formation of Li dendrites. **b** Schematics showing Li deposition on an electrode with a GO-*g*-PAM@PP separator. The GO-*g*-PAM molecular brushes with a large quantity of polar functional groups (C = O, N–H) provide high-concentration functional sites for the efficient adhesion and homogeneous distribution of Li ions at the molecular level, giving rise to dendrite-free uniform Li deposition

Nevertheless, the application of Li metal anodes has been considerably limited because of uncontrolled dendritic Li formation and the highly reactive nature of Li^13–15^. Specifically, due to the unavoidable microscopic roughness of electrode surfaces, the electrical fields near the electrode surfaces are nonuniformly distributed during cell operation, which results in an inhomogeneous distribution of Li ions. As a result, more Li will be preferentially deposited around the protuberant tips with stronger electrical fields^11,16^. Notably, this preferential deposition behavior undergoes self-amplification with gradually increasing surface roughness and tip electrical field intensity, leading to the gradual formation of Li dendrites (Fig. 1a). The resultant Li dendrites can bridge the interelectrode spaces and thus cause internal short circuits in the cell, which gives rise to obvious safety limitations on the cells^17^. On the other hand, highly reactive Li metal can irreversibly decompose the solvent and salts to instantly form a solid electrolyte interphase (SEI) protecting layer on the Li surface. Such an SEI protecting layer is too brittle to alleviate the significant volume change of Li metal during plating/stripping processes, exposing fresh Li metal underneath to the electrolyte^18^. As a result, Li dendrite growth is accelerated at the exposed sites, and Li metal and electrolyte are continuously consumed through the formation of SEI layers over repeated plating/stripping processes, giving rise to a low Coulombic efficiency and poor cycling stability. Furthermore, these issues are more severe under the high-current densities that are needed due to the increasing demand of high-power devices.

In this context, numerous efforts have been devoted to addressing these issues and unlocking the full potential of Li metal anodes. One method is to optimize the liquid electrolytes by adjusting the electrolyte compositions^19^ or adding various electrolyte additives^20–23^. These additives can react rapidly with Li metal to modify the chemical composition of SEI layers and thus improve their stability and uniformity. Nevertheless, in most cases, the in situ formed protecting layers are still not strong enough to withstand the mechanical deformation induced by dendrite growth, and stable cycling is not sustainable over a long period of time due to the consumption of such additives^24,25^. Another method is to ex situ coat the surfaces of Li metal anodes by various artificial protective layers, such as polymeric grafted skin^26^, ultrathin Al_2_O_3_ layer^27^, and LiF/PVDF-HFP composite film^28^, which offer high chemical stability and mechanical strength, thus overcoming the fragility issue. However, the artificial protective layers often result in increased polarization voltages because of their relatively high resistance^28^. Additionally, ex situ coating procedures are usually tedious and dangerous because of the highly reactive nature of Li metal, which could make large-scale industrial operations difficult.

As an important part of cells, separators play a critical role in determining cell’s electrochemical performance. It is believed that separator modification is more convenient than Li metal anode modification to achieve stable Li plating/stripping^29^. Recently, it has been proposed that high-modulus separators, such as solid-state electrolyte membranes and robust interlayers, can function as strong mechanical barriers to block dendrite growth^22,30–35^. Nevertheless, these mechanical barriers introduce the additional problem of large interfacial resistance, and the fundamental problem of uneven Li electrodeposition remains unsolved. On the other hand, a few reports have demonstrated that the use of lithiophilic separators with abundant polar functional groups can homogenize Li-ion flux on the electrode surfaces and thus promote uniform Li deposition^36–39^. Unfortunately, the mechanical strength of lithiophilic separators is rather poor and cannot readily withstand the large pressure induced by the substantial volume change of Li metal anodes^12^. Additionally, the stable plating/stripping of Li anodes can only be achieved under limited current densities (mostly no more than 2 mA cm^−2^). Therefore, it is imperative to develop advanced separators that simultaneously possess favorable mechanical strength, high ionic conductivity, and valuable lithiophilic features to boost the performance of Li metal anodes under very high current densities.

Herein, a class of functional porous bilayer composite separators is employed for the first time to regulate homogeneous Li deposition and achieve superstable Li metal anodes under ultrahigh current densities. Our advanced composite separators are facilely prepared by blade-coating two-dimensional (2D) molecular brushes (i.e., polyacrylamide-grafted graphene oxide nanosheets, GO-*g*-PAM) onto one side of commercial polypropylene (PP) separators, which is highly convenient for the large-scale production of modified separators. In the composite separators, the robust GO backbones of the molecular brushes improve the mechanical strength, while the hairy PAM chains on the GO surfaces with a large quantity of polar groups including C = O and N–H bonds provide high-concentration functional sites for the efficient adhesion and homogeneous distribution of Li ions at the molecular level. Moreover, the interspaces between the stacked 2D molecular brushes provide fast pathways for the diffusion of electrolytes. As a result, molecular-level homogeneous and fast Li ionic flux on the surface of electrodes is achieved with the porous bilayer composite separators, giving rise to dendrite-free uniform Li deposition with high Coulombic efficiencies and ultralong-term reversible Li plating/stripping under very high current densities (Fig. 1b).

## Results

### Preparation and characterization of 2D molecular brushes

**Fig. 2:**
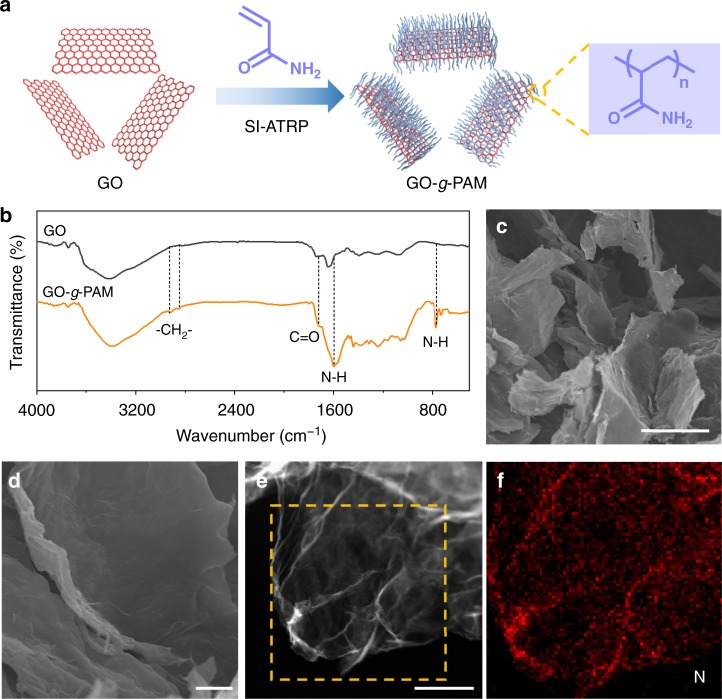
Characterization of GO-*g*-PAM molecular brushes. **a** Schematic illustration of the synthesis of GO-*g*-PAM molecular brushes: modifying GO nanosheets by α-bromoisobutyryl bromide to introduce Br-containing initiation sites and in situ grafting PAM chains from the modified surface of GO nanosheets via SI-ATRP. **b** FTIR spectra of GO and GO-*g*-PAM. **c**, **d** SEM images of GO-*g*-PAM. **e**, **f** Elemental mapping showing the molecular-level homogeneous distribution of N on GO nanosheets. Scale bars: (**c**) 20 μm, (**d**) 2 μm, and (**e**) 500 nm

The synthetic strategy of GO-*g*-PAM molecular brushes is schematically presented in Fig. 2a. The GO nanosheets are first modified by α-bromoisobutyryl bromide to introduce Br-containing initiation sites onto the surface. Afterward, hairy PAM chains are successfully grafted from the modified surface of GO nanosheets via surface-initiated atom-transfer radical polymerization (SI-ATRP), a very successful strategy for the preparation of well-defined surface polymers. The Fourier transform infrared (FTIR) spectra in Fig. 2b reveal that the PAM chains are successfully grafted from the GO nanosheets, as demonstrated by the presence of two new characteristic peaks of methylene groups at 2922 and 2853 cm^−1^ and the increased peak intensity of C = O bonds at 1720 cm^−1^ for GO-*g*-PAM. The two strong peaks at 1598 and 770 cm^−1^ for GO-*g*-PAM can be ascribed to the in-plane and out-of-plane scissoring bending vibrations of primary NH_2_ groups, respectively. The results demonstrate the presence of substantial polar functional groups, including C = O and NH_2_ in GO-*g*-PAM. The nanomorphology of GO-*g*-PAM is investigated by scanning electron microscopy (SEM). As shown in Fig. 2c, d, the as-prepared GO-*g*-PAM molecular brushes present a typical 2D nanosheet morphology. No obvious polymer particles are observed on the surface, indicating homogeneous PAM grafting to the GO surface. Elemental mapping analysis of the GO-*g*-PAM molecular brushes also reveals the molecular-level homogeneous distribution of N (Fig. 2e, f), further confirming the uniform grafting of PAM chains and the homogeneous distribution of their acylamido groups. Therefore, benefiting from the unique 2D nanosheet structure of GO-*g*-PAM, the polar functional groups of the surface PAM chains can be largely exposed, which can facilitate homogeneous Li ionic flux and deposition.

### Fabrication and characterization of functional porous bilayer composite separators

**Fig. 3:**
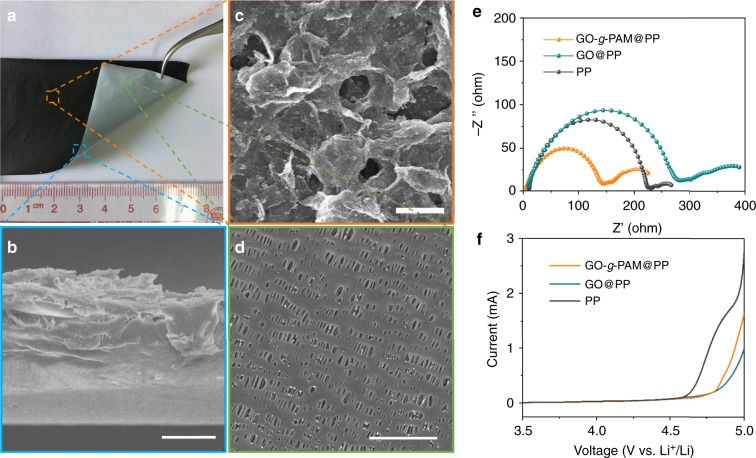
Characterization of the GO-*g*-PAM@PP separator. **a** Digital photograph of a bent GO-*g*-PAM@PP separator. **b** Cross-section and (**c**) top-view SEM images of the GO-*g*-PAM@PP separator. **d** SEM image of the uncoated side of the GO-*g*-PAM@PP separator. **e** Nyquist plots of symmetric Li|Li cells with PP, GO@PP, and GO-*g*-PAM@PP separators. **f** LSV curves of asymmetric Li|stainless-steel cells with PP, GO@PP, and GO-*g*-PAM@PP separators at a scan rate of 5 mV s^−1^. Scale bars: (**b**) 20 μm, (**c**) 5 μm, and (**d**) 1 μm

The GO-*g*-PAM molecular brushes are facilely coated onto one side of a commercial Celgard 2325 polypropylene (PP) separator with a blade to form a functional porous bilayer composite separator (GO-*g*-PAM@PP), whose functional porous coating layer results from the nanosheet interstacking of GO-*g*-PAM. As shown in Fig. 3a and Supplementary Fig. 1, the GO-*g*-PAM@PP separator can be bent or even rolled around a glass rod without any detachment. In contrast, the PAM-coated PP separator (PAM@PP) is very brittle, which leads to severe detachment and breaking of the coated PAM layer once it is slightly bent (Supplementary Fig. 2). Additionally, the atomic force microscope (AFM) Young’s modulus mappings reveal that the GO-*g*-PAM@PP separator exhibits a higher Young’s modulus than the PP and PAM@PP separators (Supplementary Fig. 3), suggestive of excellent mechanical strength enabled by the GO-*g*-PAM molecular brushes. A cross-section SEM image of GO-*g*-PAM@PP shows that the coated GO-*g*-PAM layer adheres closely to the surface of the PP layer (Fig. 3b). The SEM images in Fig. 3c, d and Supplementary Fig. 4 show that both the GO-*g*-PAM-modified layer and the PP layer have well-developed pores. The pores in the functional layer can be ascribed to the interspaces between the interstacked nanosheets (Fig. 3c). Moreover, due to their large sheet size, GO-*g*-PAM nanosheets cannot enter the pores of the original PP separator during coating. Thus, the porous structure of the PP layer is retained very well in the GO-*g*-PAM@PP (Fig. 3d and Supplementary Fig. 4). N_2_ adsorption–desorption isotherm and density functional theory (DFT) pore size distribution (PSD) curve in Supplementary Fig. 5 further indicate that the GO-*g*-PAM@PP separator has numerous mesopores and macropores with a maximum PSD peak at 54 nm. It should be noted that some large macropores may be too large and too open to cause capillary condensation and thus are not reflected in the PSD curve. Moreover, the large electrolyte uptake of the GO-*g*-PAM@PP separator also confirms its highly porous structure (Supplementary Fig. 6). These well-developed pores can provide fast pathways for Li ionic diffusion. As shown in Supplementary Fig. 7, the electrolyte can rapidly spread and permeate to the bottom side of the GO-*g*-PAM@PP separator when it is dropped onto the top GO-*g*-PAM coating.

The GO-*g*-PAM@PP separator demonstrates great affinity with the electrolyte, because of the substantial polar functional groups^22^. As shown in Supplementary Fig. 8, the contact angle of electrolyte on the GO-*g*-PAM@PP separator is almost zero, much lower than that on the PP separator (23°). This enhanced affinity with the electrolyte allows a good electrode−electrolyte contact and thus enhances the Li ionic transport near the electrode−electrolyte interface, leading to a decreased interface resistance between the Li electrode and electrolyte. More importantly, the strong lithiophilic characteristics of PAM chains also endow the GO-*g*-PAM@PP separator with a significant electrokinetic pumping feature, which can accelerate the Li-ion transport and further decrease the battery resistance^40^. As shown in Fig. 3e, the symmetric Li|Li cell with the GO-*g*-PAM@PP separator demonstrates a considerably lower interfacial impedance than those with the PP separator and GO@PP separator, verifying the enhanced Li ionic transport kinetics. The cell with the GO@PP separator has a higher interfacial impedance than that with the PP separator, indicating the slow infiltration of the liquid electrolyte through the GO layers, which further demonstrates the important role of the surface PAM chains. A linear sweep voltammetry (LSV) test suggests that the GO-*g*-PAM@PP separator is stable up to 4.7 V vs. Li^+^/Li, performance similar to that of the GO@PP separator yet slightly higher than that of the PP separator, indicating the high electrochemical stability of the GO-*g*-PAM coating (Fig. 3f). Moreover, the electrical conductivity tests reveal the electrically insulated feature of the GO-*g*-PAM@PP separator (Supplementary Fig. 9).

### Electrochemical performance of GO-*g*-PAM@PP as a separator in Li metal batteries

Benefiting from the aforementioned unique physiochemical merits, the GO-*g*-PAM@PP separator is promising for regulating Li deposition and suppressing Li dendrite growth at a molecular scale. As shown in Supplementary Fig. 10a, b, typical mossy Li in the submicron size range with nonuniform structure is clearly observed after directly plating 1 mAh cm^−2^ of Li on the Cu foils in the cells with PP and GO@PP separators, respectively. In contrast, Li metal is homogeneously deposited on the Cu foil under identical conditions when GO-*g*-PAM@PP is used as the separator, leading to the formation of a dendrite-free morphology with a considerably flat surface (Supplementary Fig. 10c). The results demonstrate that the GO-*g*-PAM can facilitate the homogeneous redistribution of the Li ions that accumulate around the protuberant tips on the Cu foil (Supplementary Fig. 11), giving rise to uniform Li deposition.

The overall electrochemical performance of Li|Cu cells with various separators is further investigated by a galvanostatic cycling test. The Li|Cu cells are first cycled from 0 to 1 V at 50 μA for five cycles to stabilize the interfaces^41^. Notably, a pair of stable lithiation and delithiation plateaus corresponding to a small areal capacity of ~0.1 mAh cm^−2^ are observed for the cell with the GO-*g*-PAM@PP separator but are absent for the cells with the PP and GO@PP separators (Supplementary Fig. 12), verifying the lithiophilic characteristics of the PAM molecular chains^34^. Thus, the Li nucleation overpotential, based on the difference between the tip voltage and the flat voltage of the first Li plating voltage profile, is only 33 mV for the GO-*g*-PAM@PP separator, much lower than those of the PP and GO@PP separators (Supplementary Fig. 13).

**Fig. 4:**
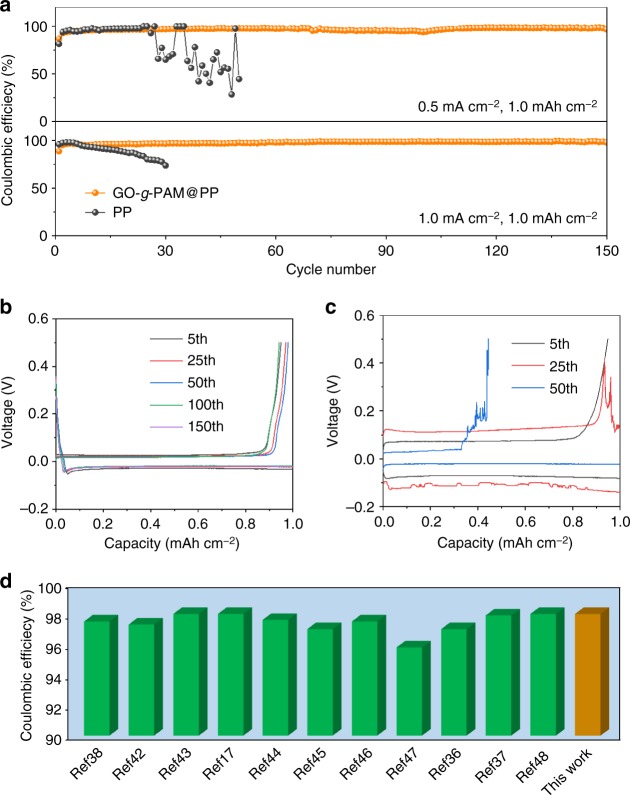
Electrochemical performance of Li|Cu cells with GO-*g*-PAM@PP and PP separators. **a** Coulombic efficiencies of Li|Cu cells with GO-*g*-PAM@PP and PP separators with a cycling capacity of 1 mAh cm^−2^ at various current densities. Voltage profiles of Li plating/stripping processes in Li|Cu cells with (**b**) GO-*g*-PAM@PP and (**c**) PP separators with a cycling capacity of 1 mAh cm^−2^ at 0.5 mA cm^−2^. **d** Average Coulombic efficiency of a Li|Cu cell with GO-*g*-PAM@PP separator tested with a current density of 1 mA cm^−2^, compared with that of previously reported asymmetric cells with Li metal anodes stabilized by various strategies

The cycling Coulombic efficiencies of the Li|Cu cells with various separators are further examined. Herein, Li metal with a total capacity of 1 mAh cm^−2^ is first plated on the Cu foil working electrodes, and then, the deposited Li is completely stripped from the Cu foils with a cutoff voltage of 0.5 V at the same current density. As shown in Fig. 4a, b, the Li|Cu cell with the GO-*g*-PAM@PP separator demonstrates a steady Coulombic efficienciy of 97% with stable plating/stripping voltage profiles for more than 150 cycles at a current density of 0.5 mA cm^−2^. In comparison, the cells with the PP and GO@PP separators display significantly inferior electrochemical cycling properties with severely fluctuating plating/stripping voltage profiles and Coulombic efficiencies during the cycles (Fig. 4a, c and Supplementary Fig. 14); these fluctuations can be ascribed to the continuous breakdown/repair of SEI, which consumes both Li and electrolyte. In addition, the Li|Cu cell with the GO-*g*-PAM@PP separator shows a smaller and more stable voltage hysteresis than those with the PP and GO@PP separators (Supplementary Fig. 15). The cell with the GO-*g*-PAM@PP separator still maintains an average Coulombic efficiency of 98% over 150 cycles under an increased current density of 1 mA cm^−2^, which is superior to those of the cells with PP and GO@PP separators and favorably comparable with those of previously reported asymmetric cells with Li metal anodes stabilized by various strategies (Fig. 4a, d and Supplementary Fig. 16)^17,36–38,42–48^. To further demonstrate the unique merits of GO-*g*-PAM molecular brushes, a Li|Cu cell with a separator coated with a mixture of GO and PAM, denoted as GO/PAM@PP, is also investigated under identical conditions. As shown in Supplementary Fig. 16, the GO-*g*-PAM@PP separator greatly outperforms the GO/PAM@PP separator for Li plating/stripping. The results demonstrate that the molecular-level uniform distribution of polar functional groups enabled by the GO-*g*-PAM molecular brushes is crucial for achieving dendrite-free deposition of Li metal, which results in stable SEI layers with minimized side reactions between the deposited Li and the electrolyte.

**Fig. 5:**
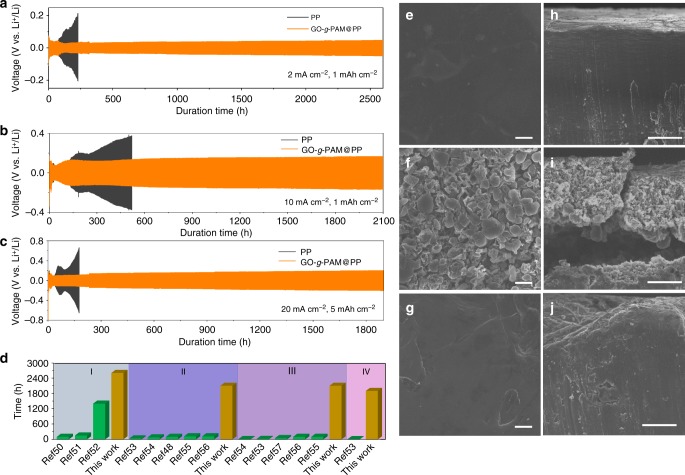
Electrochemical performance of symmetric Li|Li cells with GO-*g*-PAM@PP and PP separators. Voltage–time profiles of Li plating/stripping processes with a cycling capacity of (**a**, **b**) 1 and (**c**) 5 mAh cm^−2^ at (**a**) 2, (**b**) 10, and (**c**) 20 mA cm^−2^ in symmetric Li|Li cells with GO-*g*-PAM@PP and PP separators. **d** Comparison of the cycle life of symmetric Li|Li cells with GO-*g*-PAM@PP separators and that of previously reported Li metal anodes stabilized by various strategies at the same current densities: (I) 2, (II) 5, (III) 10, and (IV) 20 mA cm^−2^. All symmetric cells were cycled at a cycling capacity of 1 mAh cm^−2^, except that the cycling capacities were 0.4 mAh cm^−2^ for ref. ^51^ and 2 mAh cm^−2^ for ref. ^52^ at 2 mA cm^−2^, and 5 mAh cm^−2^ for this work at 20 mA cm^−2^. **e**–**g** Top-view and (**h**–**j**) cross-section SEM images of (**e**, **h**) a fresh Li metal anode and Li metal anodes assembled with (**f**, **i**) PP and (**g**, **j**) GO-*g*-PAM@PP separators after 100 cycles. Scale bars: (**e**–**g**) 10 μm and (**h**–**j**) 50 μm

To further investigate the advantage of the GO-*g*-PAM@PP separator in the cycling stability of Li metal anodes, symmetric Li|Li cells with various separators are fabricated. As shown in Fig. 5a, when the cycling capacity is 1 mAh cm^−2^, the cell with the GO-*g*-PAM@PP separator delivers an excellent cycling stability with stable voltage plateaus for over 2600 h at a current density of 2 mA cm^−2^. In sharp contrast, the cell with the GO@PP separator exhibits a much larger voltage hysteresis (Supplementary Fig. 17); a gradual increase in voltage hysteresis is observed for the cell with the PP separator after cycling for only 73 h (Fig. 5a), which can be ascribed to the electrical disconnection and depletion of electrolyte resulting from the repeated growth/corrosion of Li dendrites and continuous consumption of electrolyte^49^. Even at very high current densities of 5 and 10 mA cm^−2^, the cells with the GO-*g*-PAM@PP separators still exhibit long-term stability for over 2100 h, greatly outperforming the cells with the PP separators (Fig. 5b and Supplementary Fig. 18). Remarkably, when the areal capacity is substantially increased to 5 mAh cm^−2^, which is much higher than that of current commercial batteries (3 mAh cm^−2^), stable Li plating/stripping of over 1900 h can still be achieved for the cell with the GO-*g*-PAM@PP separator at an extremely high current density of 20 mA cm^−2^, whereas the cell with the PP separator can only be stably cycled for no more than 36 h (Fig. 5c). The significant differences in cycle stability strongly demonstrate the superiority of GO-*g*-PAM@PP separator for repeated plating and stripping of Li. To the best of our knowledge, such an ultralong lifespan is superior to those of all previously reported Li anodes stabilized by various strategies under similar test conditions, which exhibit significantly decreased cycling performance when the current density is larger than 2 mA cm^−2^ (Fig. 5d)^48,50–57^. Moreover, the electrochemical performance of our GO-*g*-PAM@PP separators could be further optimized by tuning the structure of GO-*g*-PAM, e.g., the molecular weight of the PAM chains by changing the polymerization time (Supplementary Fig. 19).

It should be noted that the cells with GO-*g*-PAM@PP separators exhibit slight voltage fluctuations with a slightly larger voltage hysteresis than that of cells with PP separators in the initial cycles (Supplementary Fig. 20). This difference could be ascribed to SEI formation on the surfaces of GO-*g*-PAM molecular brushes that directly contact the Li foils (Supplementary Fig. 12a)^58,59^. After the initial few cycles, the SEI is mature and stabilized, and thus the cells with GO-*g*-PAM@PP separators begin to demonstrate a flatter plateaus and smaller average voltage hysteresis in later long cycles. For further evidence, the voltage–time profile of a symmetric Li|Li@Cu cell with a prestabilized GO-*g*-PAM@PP separator is also provided. As shown in Supplementary Fig. 21, this cell demonstrates much more stable voltage plateaus with reduced overpotentials at 5 mA cm^−2^.

The nanomorphologies of Li metal anodes after symmetric cell cycles are investigated to further clarify the effect of the GO-*g*-PAM molecular brushes on the suppression of Li dendrites. As shown in Fig. 5e, h, the fresh Li metal anode presents a dense structure with a smooth surface. However, for the cell with the PP separator, the Li metal anode displays obvious wire-shaped Li dendrites after the first cycle, and loosely stacked mossy Li with a highly porous structure is formed after 10 cycles; this latter morphology becomes more significant after 50 and 100 cycles (Fig. 5f, i, and Supplementary Fig. 22). In sharp contrast, after coating the PP separator with GO-*g*-PAM molecular brushes, uniform and dense Li deposition is realized and no dendrite formation is observed on the anode surface after the first cycle (Supplementary Fig. 23a, b). Importantly, the Li metal anode still retains a relatively dense and compact structure with a dendrite-free flat surface even after 50 and 100 cycles, highlighting the advantages of GO-*g*-PAM molecular brushes for favorable dendrite-free Li plating/stripping behavior (Fig. 5g, j, and Supplementary Fig. 23c–f).

**Fig. 6:**
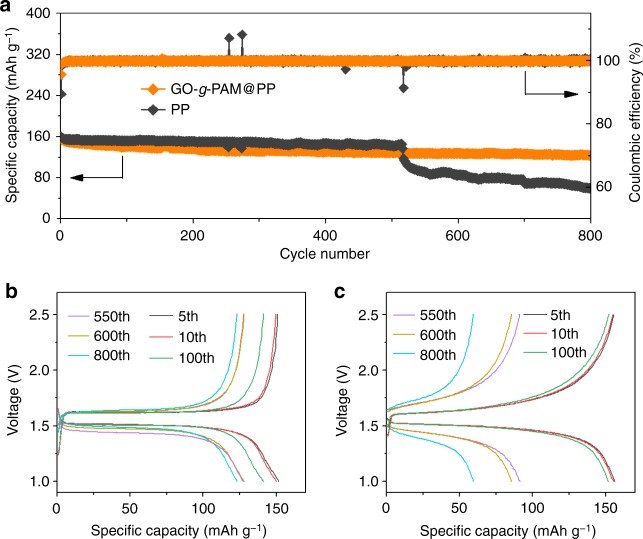
Electrochemical performance of Li|Li_4_Ti_5_O_12_ cells with GO-*g*-PAM@PP and PP separators. **a** Long-term cycling stability of Li|Li_4_Ti_5_O_12_ cells with GO-*g*-PAM@PP and PP separators at a current density of 3 C (1 C = 175 mA g^−1^). Galvanostatic charge–discharge profiles of Li|Li_4_Ti_5_O_12_ cells with (**b**) GO-*g*-PAM@PP and (**c**) PP separators at 3 C

To demonstrate the potential of the GO-*g*-PAM@PP separator in practical Li metal batteries, the electrochemical performance of Li|Li_4_Ti_5_O_12_ cells with GO-*g*-PAM@PP and PP separators is also investigated. As expected, the Li|Li_4_Ti_5_O_12_ cell with the GO-*g*-PAM@PP separator demonstrates better cycling performance than that with the PP separator. Specifically, the cell with the GO-*g*-PAM@PP separator delivers a specific capacity of 160 mAh g^−1^ with a Coulombic efficiency of 96% at the first cycle and retains 77% of the initial capacity with a steady Coulombic efficiency of nearly 100% over 800 cycles (Fig. 6a, b). In comparison, the Coulombic efficiency of the Li|Li_4_Ti_5_O_12_ cell with the PP separator during the first cycle is only 90%, and the efficiency exhibits occasional fluctuations during the cycles. In addition, the capacity of the Li|Li_4_Ti_5_O_12_ cell with the PP separator suddenly degrades after 515 cycles, accompanied by a significantly increased overall overpotential (Fig. 6a, c). The results can be ascribed to the increasingly thicker SEI layer and “drying-out” of the cell caused by the continuous reaction of electrolyte with the uncontrollably deposited Li dendrites. The Li|Li_4_Ti_5_O_12_ cell with the GO-*g*-PAM@PP separator exhibits a slightly larger capacity decay than that of the cell with the PP separator in the initial cycles, which could be ascribed to the irreversible formation of SEI films in the initial cycles on the surfaces of the GO-*g*-PAM molecular brushes that directly contact the Li foils (Supplementary Fig. 12a).

## Discussion

Uniform and stable Li deposition is regulated by employing 2D molecular brush-functionalized porous bilayer composite separators, which are facilely obtained by coating the GO-*g*-PAM molecular brushes on commercial PP separators with an upscalable blade-coating procedure. Benefiting from the abundant polar functional groups of hairy PAM chains, the interconnected interspaces between the stacked 2D molecular brushes, and the robust mechanical strength of GO nanosheets, the functional porous bilayer composite separators not only possess excellent mechanical strength but also facilitate molecular-level homogeneous and fast Li ionic flux on the surfaces of electrodes. These unique features of the functional porous bilayer composite separators enable dendrite-free uniform Li deposition with high Coulombic efficiency at Li metal anodes as well as ultralong-term reversible Li plating/stripping at ultrahigh current densities.

## Methods

### Synthesis of GO–Br

In a typical synthesis process, 100 mg of GO (purchased from Hangzhou Gaoxi Technology Co., Ltd., China) dispersed in 190 mL of N,N-dimethylformamide (DMF) was added to a three-necked round-bottom flask (250 mL) and sonicated for 0.5 h, followed by adding 1.3 mL of triethanolamine. After cooling to 0 °C and purging with N_2_ for 30 min, a mixed solution of α-bromoisobutyryl bromide (0.857 mL) and DMF (10 mL) was added dropwise for 0.5 h. The reaction was carried out under stirring at 0 °C for 2 h, followed by 30 °C for 24 h. The resulting GO–Br was then centrifuged at 12,000 rpm, washed with deionized water three times, and redispersed in 100 mL of deionized water.

### Synthesis of GO-*g*-PAM molecular brushes

Typically, 2,2′-bipyridine (0.0413 g) and acrylamide (0.9467 g) were first added to the above GO–Br aqueous dispersion and stirred in a Schlenk flask under a N_2_ atmosphere for 30 min. A total of 0.0187 g of CuBr was then added to the mixture, and the solution was continually stirred under a N_2_ atmosphere for another 10 min. Afterwards, the reaction was carried out at 70 °C for 12 h. The polymerization was stopped by opening the flask and exposing the catalyst to air. The resulting GO-*g*-PAM was centrifuged at 12,000 rpm for 10 min, washed with deionized water three times, and then freeze-dried for 24 h. GO-*g*-PAM molecular brushes with different molecular weights of PAM chains were also prepared by controlling the polymerization times.

### Synthesis of PAM

A total of 7.815 g of acrylamide, 0.1563 g of 2,2′-azobis-(2-methylpropionitrile), 45 mL of ethanol, and 45 mL of acetone were added into a three-necked round-bottom flask (150 mL) under a N_2_ atmosphere with stirring for 30 min. The flask was then immersed in a water bath at 60 °C for 3 h. Afterwards, the precipitates were filtered and washed with ethanol three times, followed by vacuum drying at 40 °C overnight.

### Fabrication of functional porous bilayer composite separators

Typically, GO-*g*-PAM and polyvinylidene difluoride (PVDF) binder with a mass ratio of 80:20 were mixed in N-methyl-2-pyrrolidinone (NMP) and then coated onto one side of a commercial PP separator (Celgard 2325) with a doctor blade, followed by vacuum drying at 50 °C overnight. After that, the GO-*g*-PAM@PP separators were punched into disks with a diameter of 19 mm. Control composite separators, including GO@PP, PAM@PP, and GO/PAM@PP, were prepared by replacing GO-*g*-PAM with GO, PAM, and the mixture of GO and PAM with the same PAM content as GO-*g*-PAM, respectively,  under identical fabrication conditions.

### Material characterization

The nanomorphologies were visualized by field-emission scanning electron microscopy (FESEM, S-4800). Elemental mapping was achieved by transmission electron microscopy (TEM, Tecnai G2 Spirit). A Micromeritics ASAP 2020 surface area and porosity analyzer was used to analyze the pore structure of the separators, and the PSD was obtained by using a DFT model. FTIR spectra were recorded at room temperature on a Bruker Equinox 55 FTIR spectroscope. Contact angle measurements were conducted by using a KRUSS DSA100 machine. The Young’s moduli of the separators were measured by AFM (Bruker Dimension Fastscan Bio) in peak force quantitative nanomechanics mode and analyzed by the Derjaguin–Muller–Toporov model.

### Electrochemical measurements

All electrochemical measurements were conducted by using CR2032 coin cells at room temperature. The electrolyte used for Li deposition was 30 µL of 1.0 M LiTFSI in a mixture of 1,3-dioxalane and dimethyl ether (1:1 by volume) with 1 wt% LiNO_3_ additive, while the electrolyte used for Li|Li_4_Ti_5_O_12_ cells was 1.0 M LiPF_6_ in a 50:50 (w/w) mixture of ethylene carbonate and diethyl carbonate. LSV measurements were collected with Li foil as the counter electrode, a stainless-steel disc as the working electrode, and the tested separator sandwiched between the electrodes with the coated side toward the Li foil. Electrochemical impedance spectra were measured with symmetric Li|Li cells by applying an AC amplitude of 5 mV over a frequency range from 0.01 to 10^5^ Hz. The asymmetric Li|Cu cells were assembled with the coated side of the composite separators toward the Cu foils, and then cycled from 0 to 1 V at 50 μA for five cycles to stabilize the interface. The Coulombic efficiency was calculated as the ratio of the stripping versus the plating capacity. The symmetric Li|Li cells were assembled with two Li foils as the working electrode and counter electrode, respectively, and PP or GO-*g*-PAM@PP separators as the separators. To prepare the Li_4_Ti_5_O_12_ electrode, Li_4_Ti_5_O_12_, acetylene black, and PVDF at a weight ratio of 7:2:1 were mixed in NMP to form a homogeneous slurry, and then rigorously pasted on a Cu foil. After drying at 60 °C under vacuum for 12 h, the Li|Li_4_Ti_5_O_12_ cells were assembled with PP or GO-*g*-PAM@PP separators. The cells were galvanostatically cycled between 1.0 and 2.5 V at 3 C (1 C = 175 mA g^−1^).

## Supplementary information

Supplementary Information

## Data Availability

The authors declare that all the data supporting the findings of this study are available within the article and its Supplementary Information or from the corresponding author upon reasonable request.
