# Supplementary material for: Two-dimensional molecular brush-functionalized porous bilayer composite separators toward ultrastable high-current density lithium metal anodes
Source: Nat Commun. 2019 Mar 25;10:1363. doi: 10.1038/s41467-019-09211-z (PMC6433896; doi:10.1038/s41467-019-09211-z)
Supplement: Supplementary file 1 — Supplementary Information [file 41467_2019_9211_MOESM1_ESM.pdf]

Supplementary Information

Two-dimensional molecular brush-functionalized porous bilayer composite separators toward ultrastable high-current density lithium metal anodes

Li et al.

Supplementary Figures

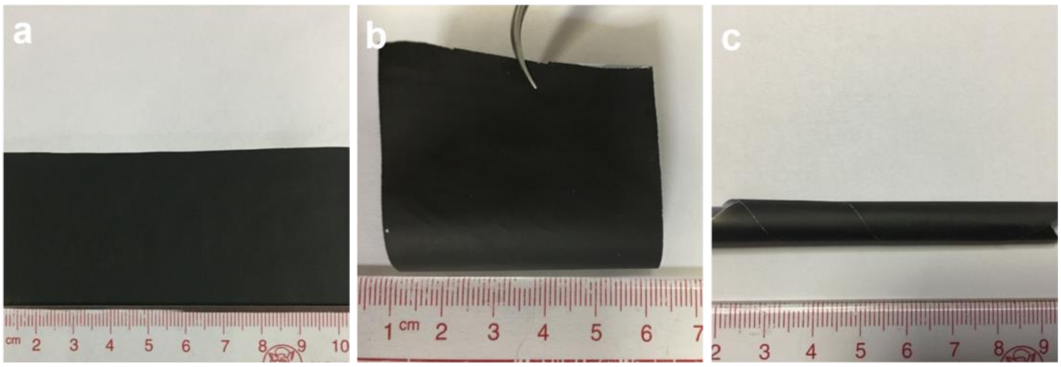

Supplementary Figure 1 | Photographs of the GO-*g*-PAM@PP separator. (a) Unfolded, (b) bent, and (c) rolled.

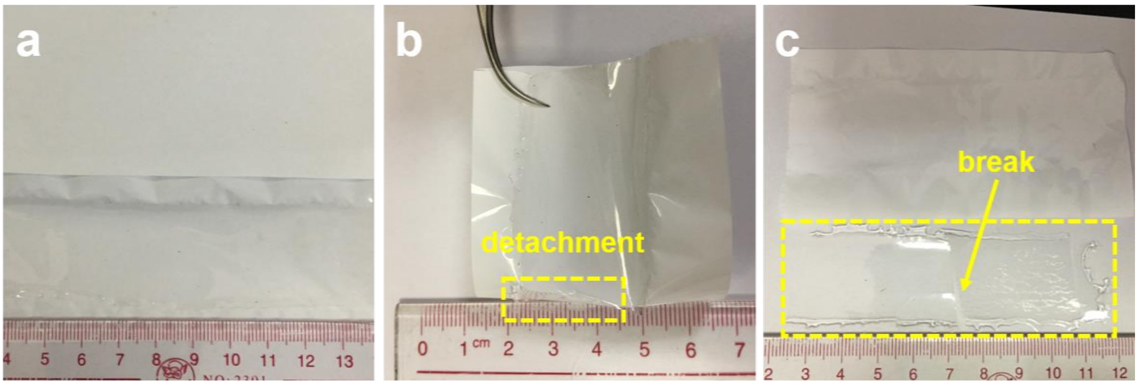

Supplementary Figure 2 | Photographs of the PAM@PP separator. Detachment and break of the PAM coating can be observed.

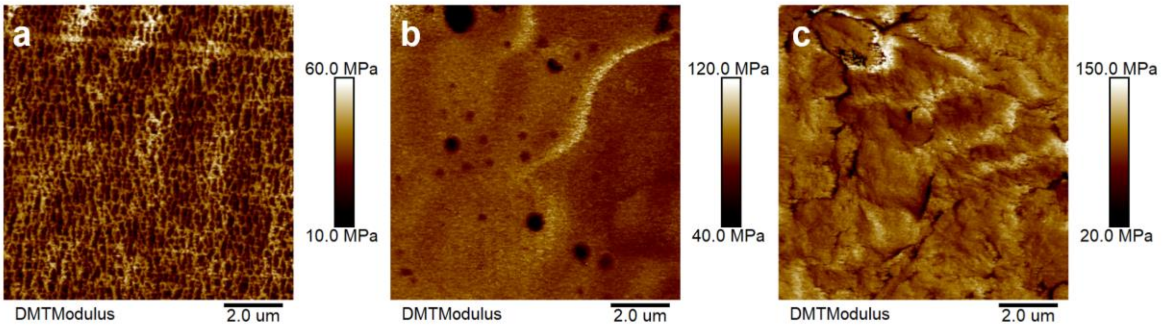

Supplementary Figure 3 | Mechanical strength of different separators. AFM Young's modulus mappings of (a) PP, (b) PAM@PP, and (c) GO-*g*-PAM@PP separators. The PAM@PP and GO-*g*-PAM@PP separators were tested on the surfaces of their PAM and GO-*g*-PAM coatings, respectively.

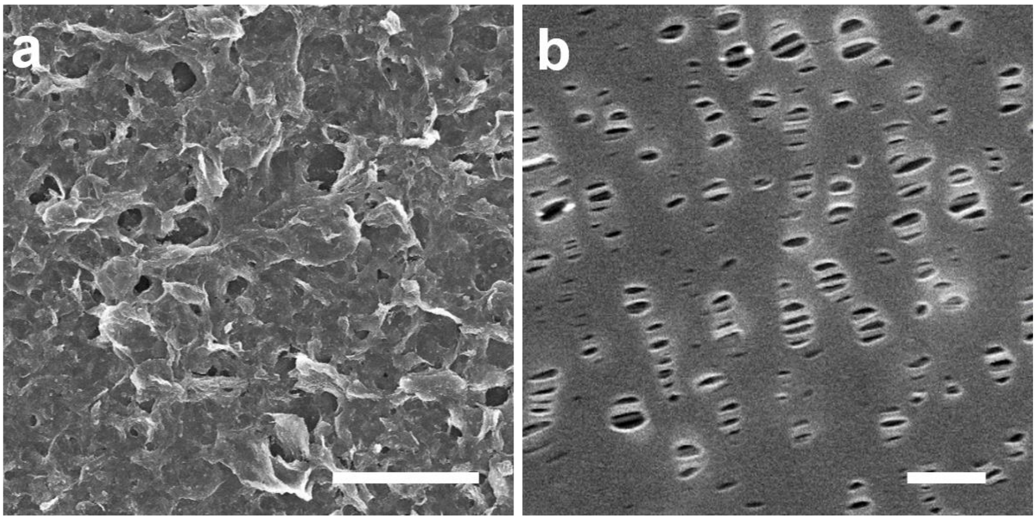

Supplementary Figure 4 | SEM images of the (a) coated and (b) uncoated side of the GO-*g*-PAM@PP separator. Scale bars, (a) 20 μm and (b) 500 nm.

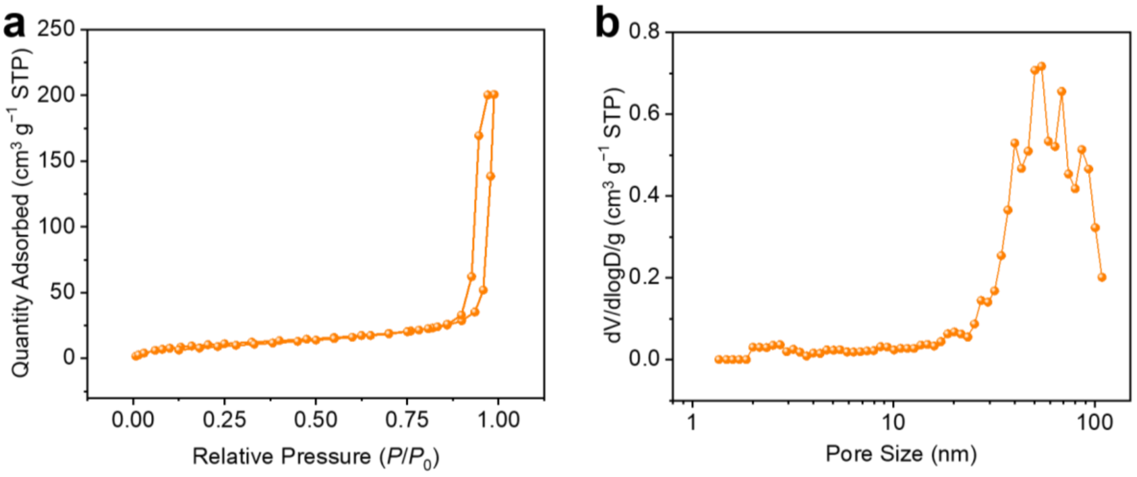

Supplementary Figure 5 | Porous structure of the GO-*g*-PAM@PP separator. (a) N_2 adsorption–desorption isotherm and (b) pore size distribution curve of GO-*g*-PAM@PP separator.

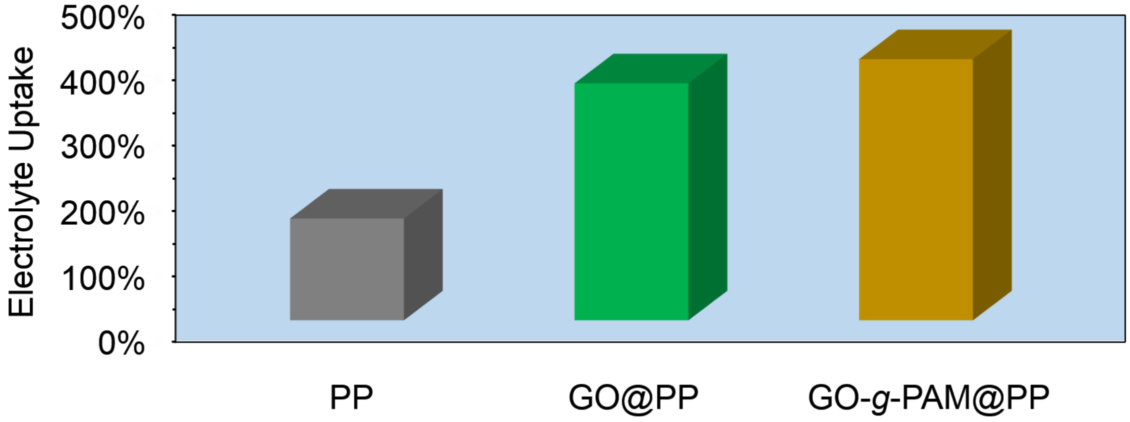

Supplementary Figure 6 | Electrolyte uptake abilities of the PP, GO@PP, and GO-*g*-PAM@PP separators.

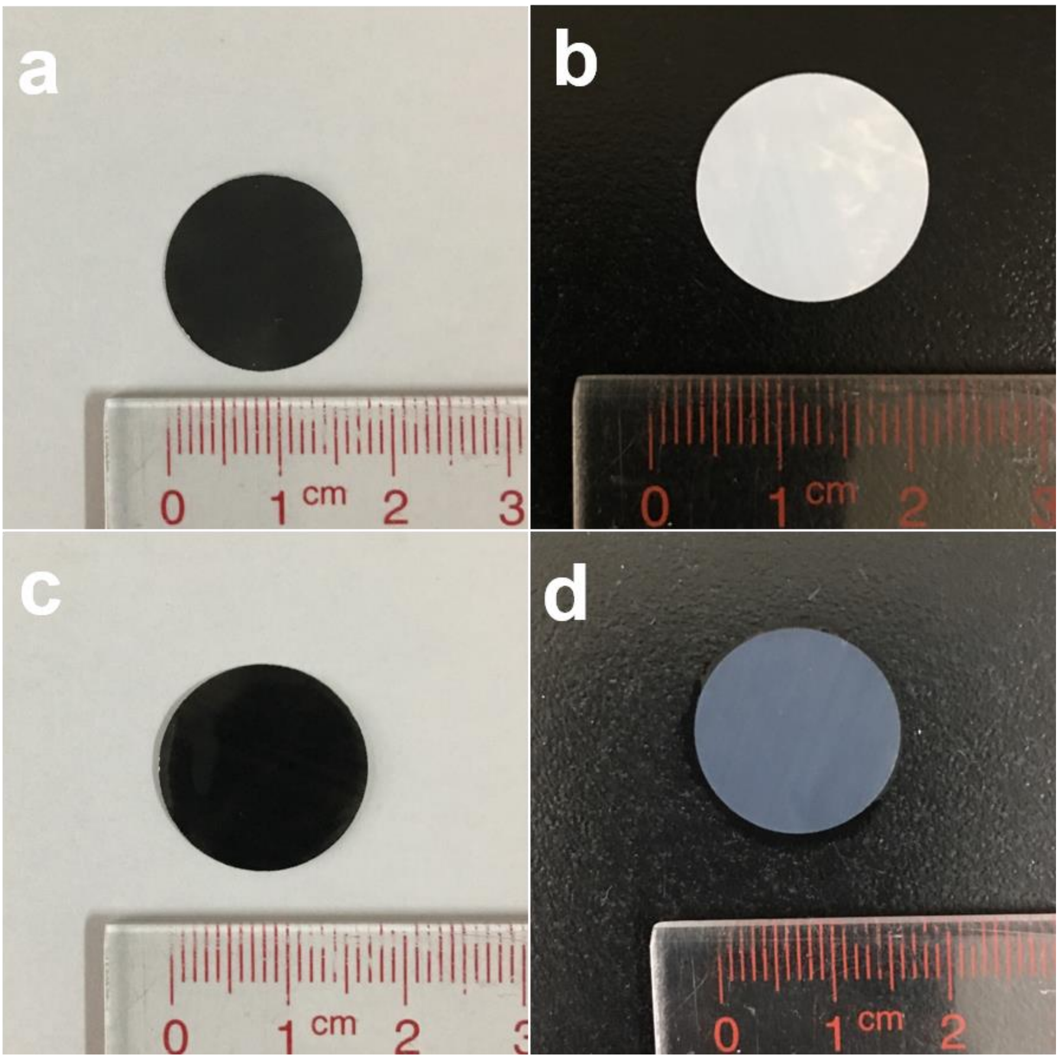

Supplementary Figure 7 | Digital photographs of the GO-*g*-PAM@PP separator. (a, c) Coated and (b, d) uncoated side of the GO-*g*-PAM@PP separator (a, b) before and (c, d) after dropping electrolyte onto the GO-*g*-PAM coating.

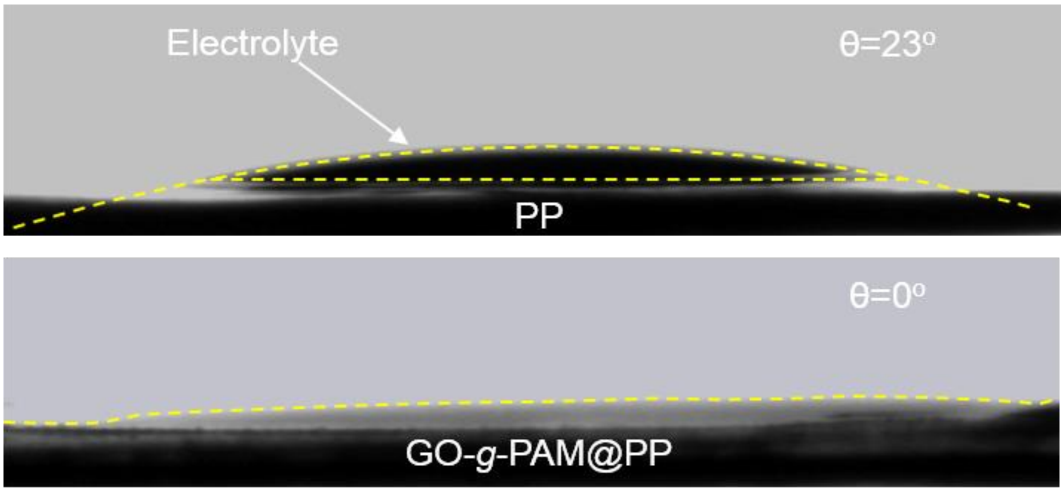

Supplementary Figure 8 | Contact angle of electrolyte on the PP and GO-*g*-PAM@PP separators.

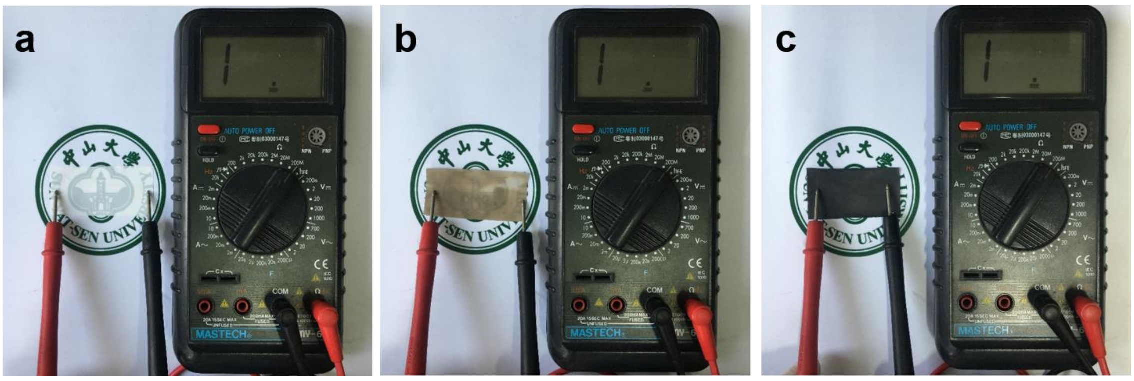

Supplementary Figure 9 | Electrical conductivity of different separators. Electrical conductivity measurements for (a) PP, (b) GO@PP, and (c) GO-*g*-PAM@PP separators by a multimeter within a measurement range of 0-200 MΩ, suggesting their electrically insulated features.

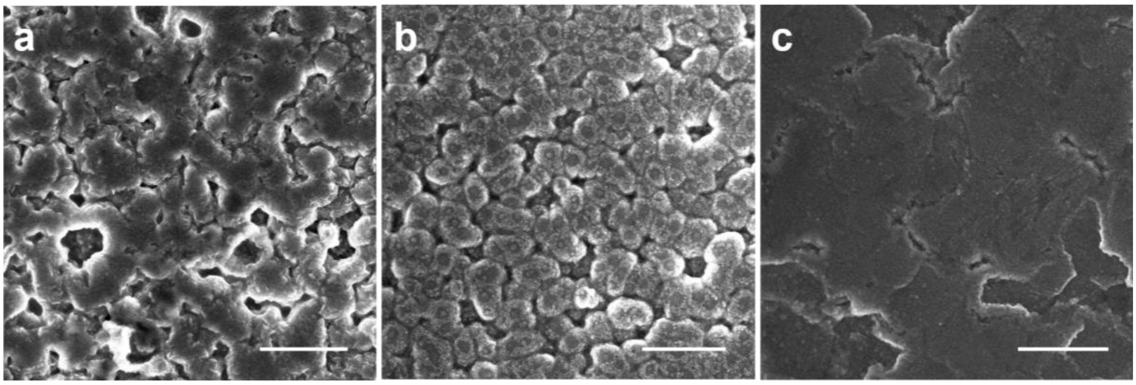

Supplementary Figure 10 | SEM images of Li deposited on Cu foils with (a) PP, (b) GO@PP, and (c) GO-*g*-PAM@PP separators. The scale bars are 10 μm.

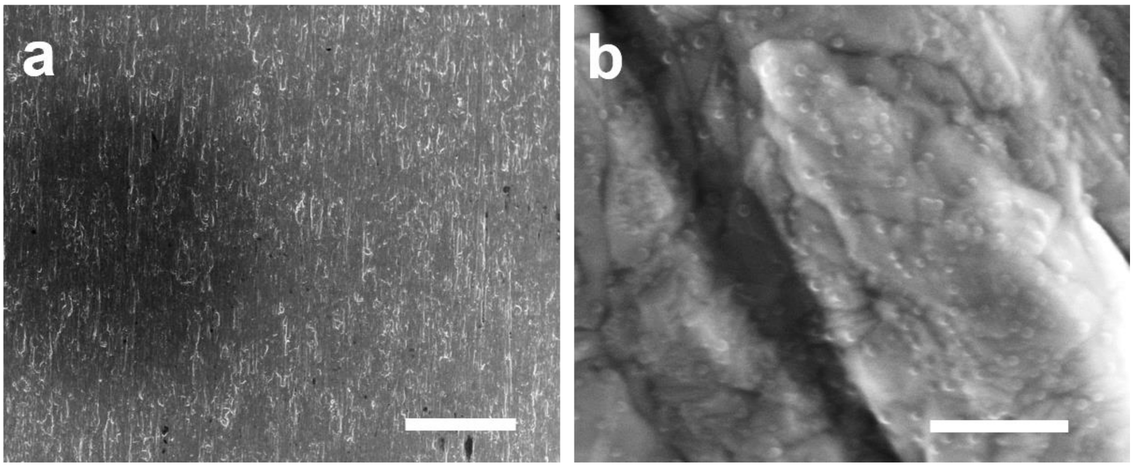

Supplementary Figure 11 | SEM images of Cu foils. Scale bars, (a) 50 μm and (b) 500 nm.

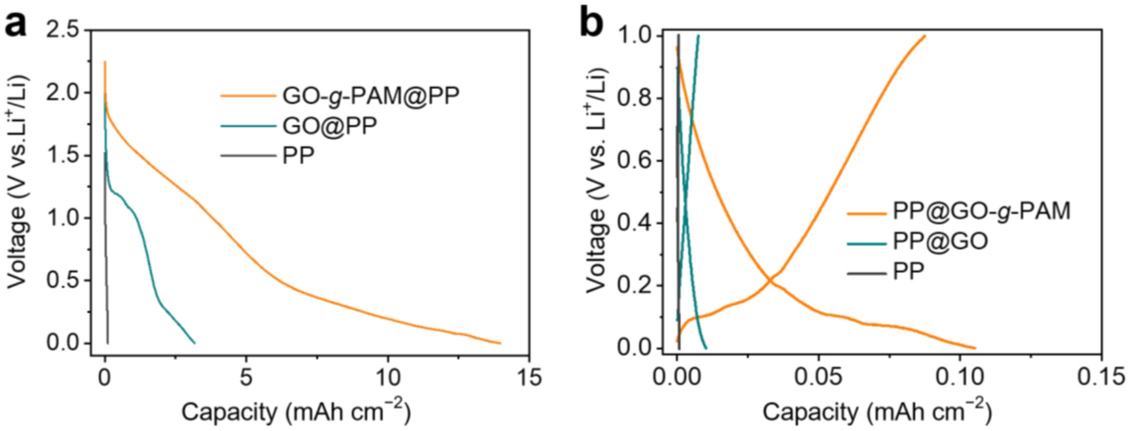

Supplementary Figure 12 | Galvanostatic charge–discharge profiles of Li|Cu cells with PP, GO@PP, and GO-*g*-PAM@PP separators at the (a) 1st and (b) 5th cycles from 0 to 1 V at 50 μA. The first discharge curve reveals that SEI films could be formed in the cell with the GO-*g*-PAM@PP separator.

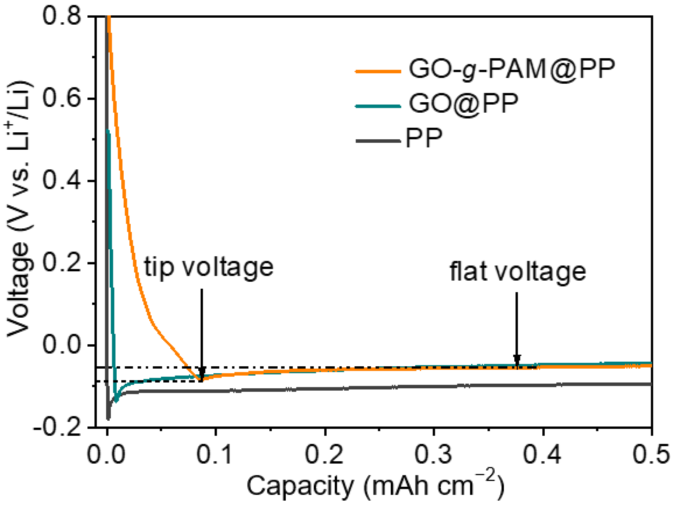

Supplementary Figure 13 | Voltage profiles of Li plating on Cu foils with PP, GO@PP, and GO-*g*-PAM@PP separators at 0.5 mA cm^{-2}.

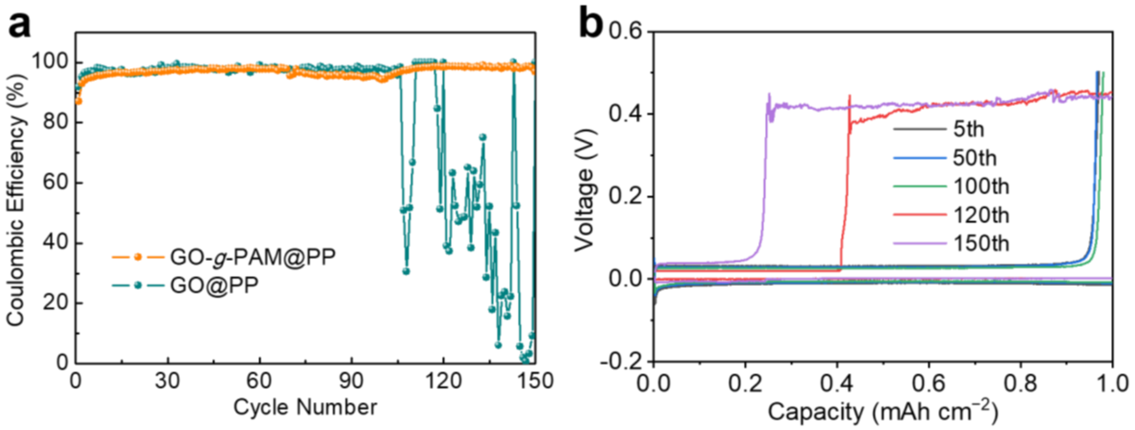

Supplementary Figure 14 | Electrochemical performance of the Li|Cu cell with the GO@PP separator. (a) Coulombic efficiencies of Li|Cu cells with GO-*g*-PAM@PP and GO@PP separators with a cycling capacity of 1 mAh cm^{-2} at 0.5 mA cm^{-2}. (b) Voltage profiles of Li plating/stripping processes in Li|Cu cells with GO@PP separators with a cycling capacity of 1 mAh cm^{-2} at 0.5 mA cm^{-2}.

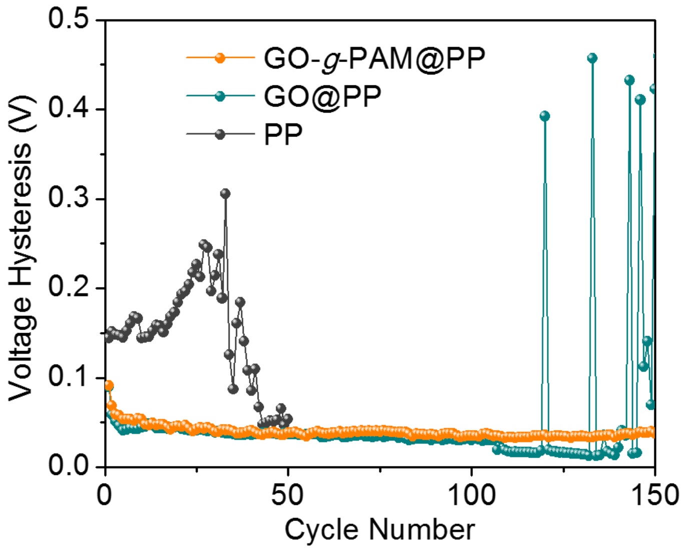

Supplementary Figure 15 | Voltage hysteresis of Li metal plating/stripping in Li|Cu cells with GO-*g*-PAM@PP, GO@PP, and PP separators.

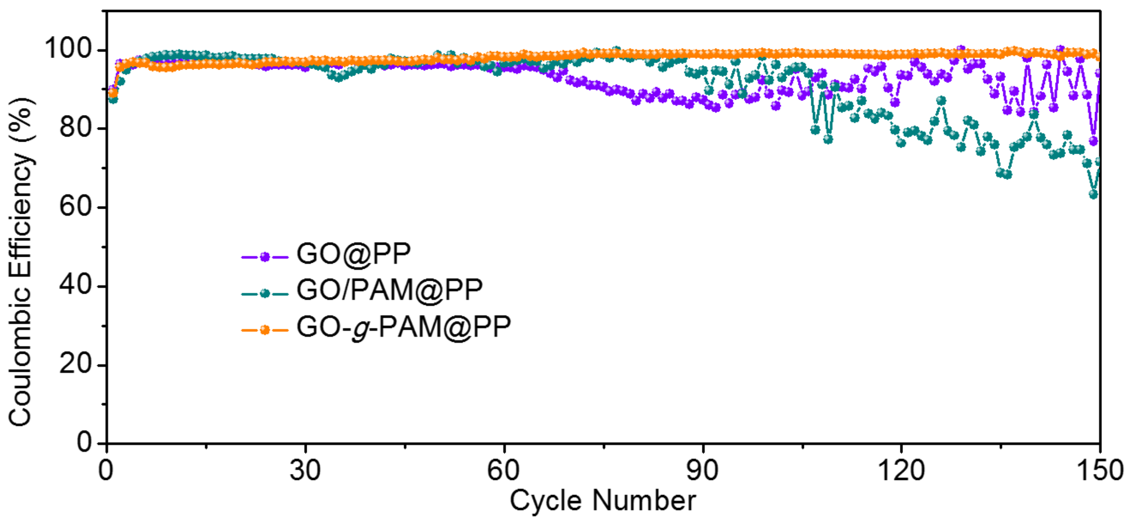

Supplementary Figure 16 | Coulombic efficiencies of Li|Cu cells with GO@PP, GO/PAM@PP and GO-*g*-PAM@PP separators with a cycling capacity of 1.0 mAh cm^{-2} at 1.0 mA cm^{-2}.

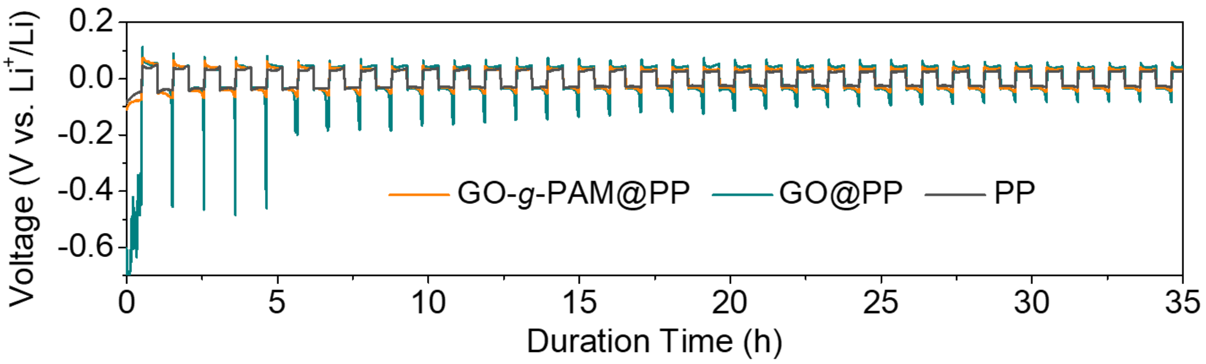

Supplementary Figure 17 | Voltage–time profiles of Li plating/stripping processes with a cycling capacity of 1 mAh cm^{-2} at 2 mA cm^{-2} in symmetric Li|Li cells with GO-*g*-PAM@PP, GO@PP and PP separators.

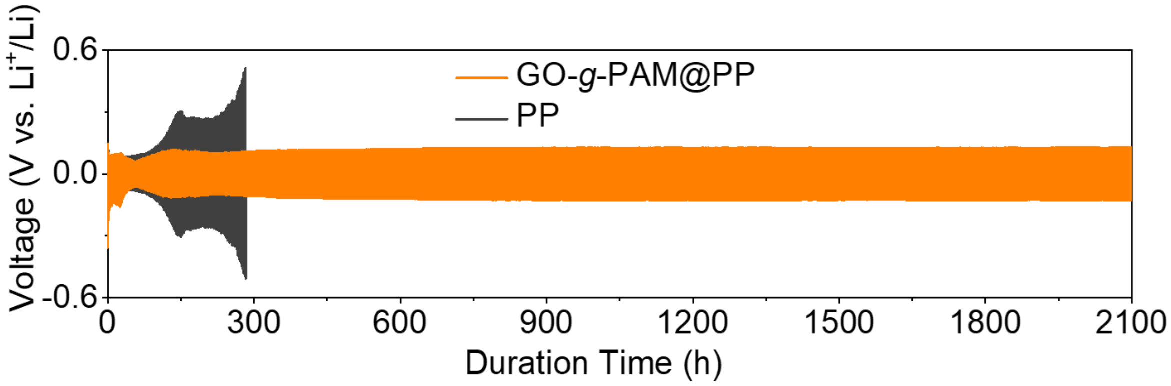

Supplementary Figure 18 | Voltage–time profiles of Li plating/stripping processes with a cycling capacity of 1 mAh cm^{-2} at 5 mA cm^{-2} in symmetric Li|Li cells with GO-*g*-PAM@PP and PP separators.

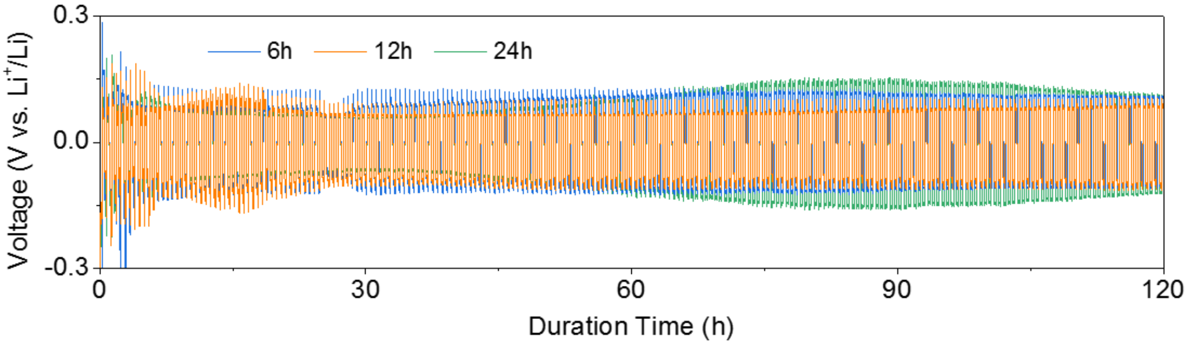

Supplementary Figure 19 | Voltage–time profiles of Li plating/stripping processes with a cycling capacity of 5 mAh cm^{-2} at 20 mA cm^{-2} in symmetric Li|Li cells with PP separators coated with GO-*g*-PAM molecular brushes prepared with different polymerization times.

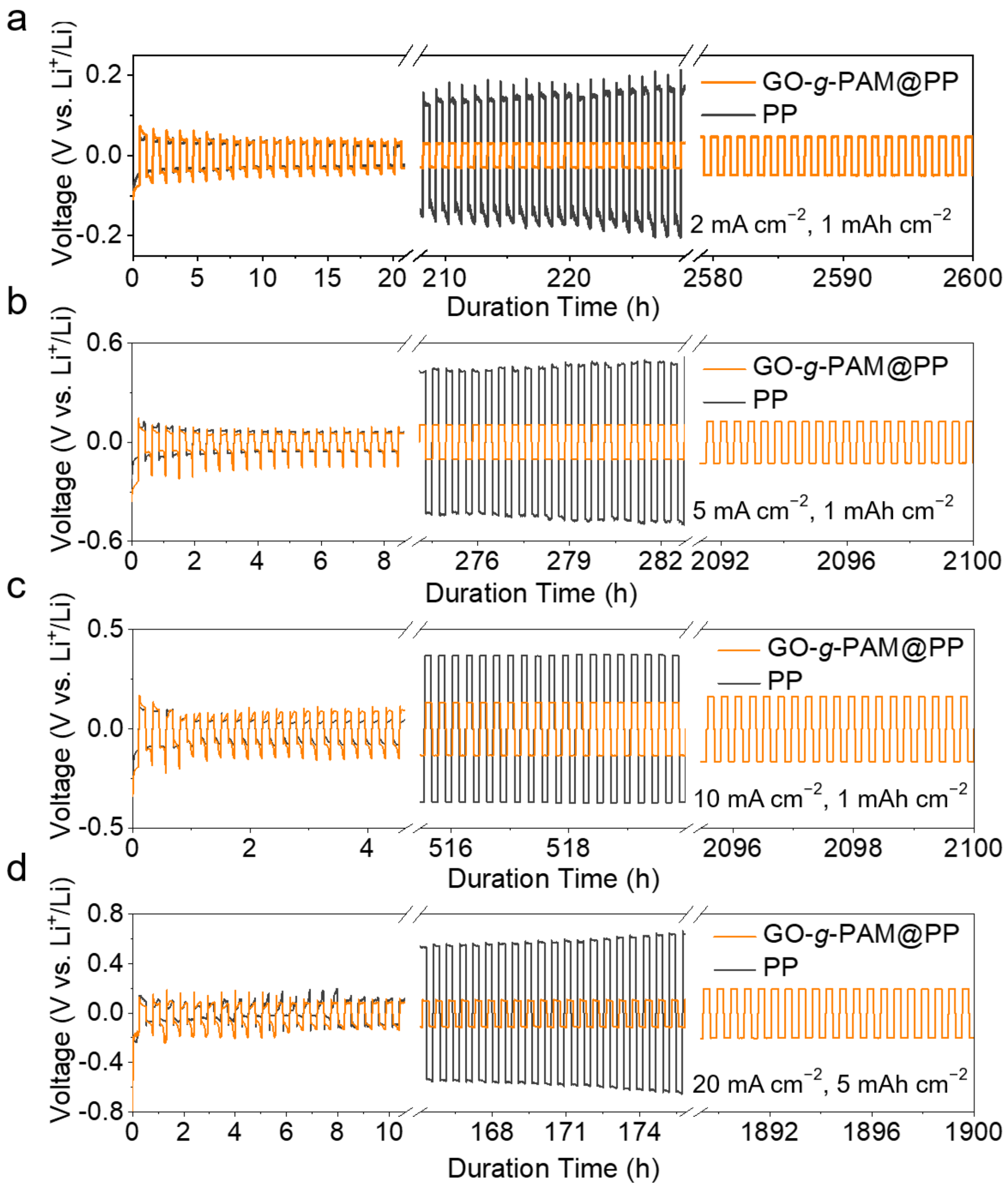

Supplementary Figure 20 | Voltage–time profiles for the initial and last cycles of symmetric Li|Li cells with GO-*g*-PAM@PP and PP separators with a cycling capacity of (a-c) 1 and (d) 5 mAh cm^{-2} at (a) 2, (b) 5, (c) 10, and (d) 20 mA cm^{-2}.

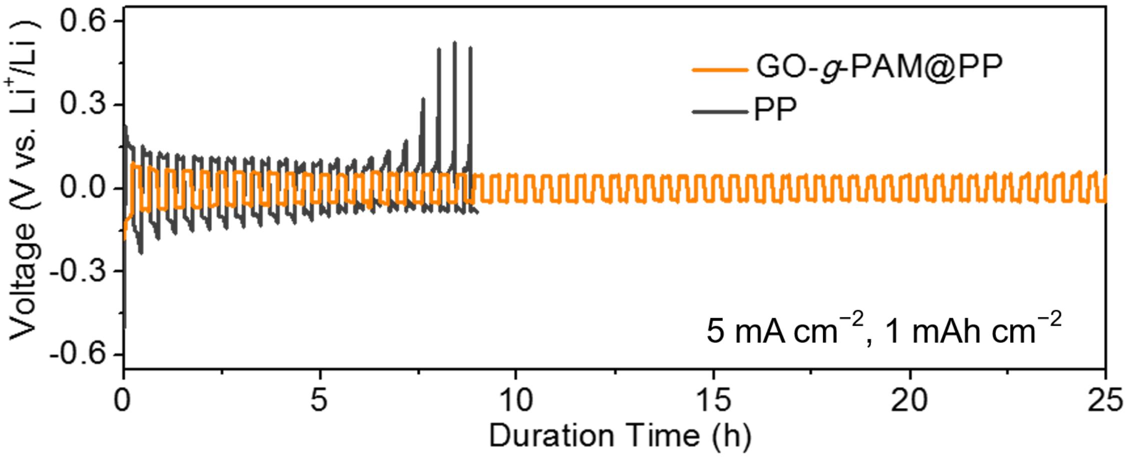

Supplementary Figure 21 | Voltage–time profiles of Li plating/stripping processes with a cycling capacity of 1 mAh cm^{-2} at 5 mA cm^{-2} in Li|Li@Cu symmetric cells with PP and GO-*g*-PAM@PP separators. The Li|Li@Cu symmetric cells were obtained by plating metallic Li onto Cu foils with a capacity of 3 mAh cm^{-2} after cycling the Li|Cu cells from 0 to 1 V at 50 µA for 5 cycles to stabilize the interface.

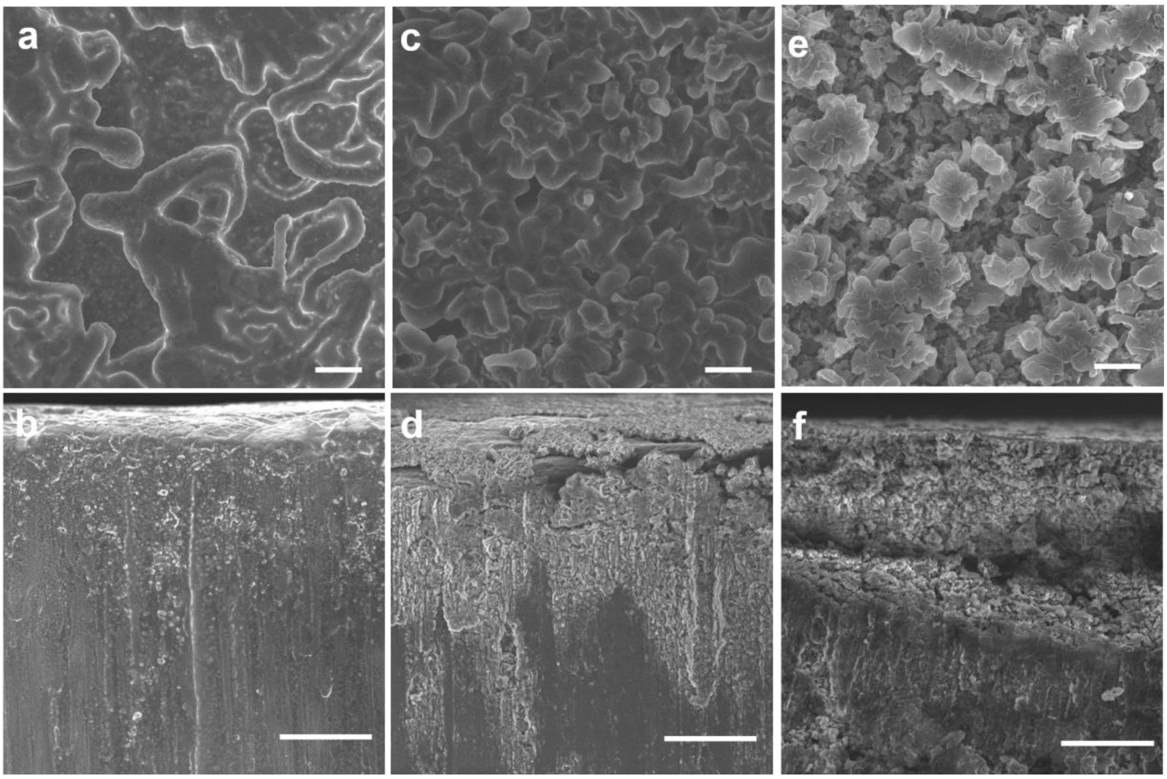

Supplementary Figure 22 | (a, c, e) Top-view and (b,d,f) cross-section SEM images of Li metal anodes assembled with PP separators after (a, b) 1, (c, d) 10, and (e, f) 50 cycles. Scale bars, (a, c, e) 10 μm and (b, d, f) 50 μm.

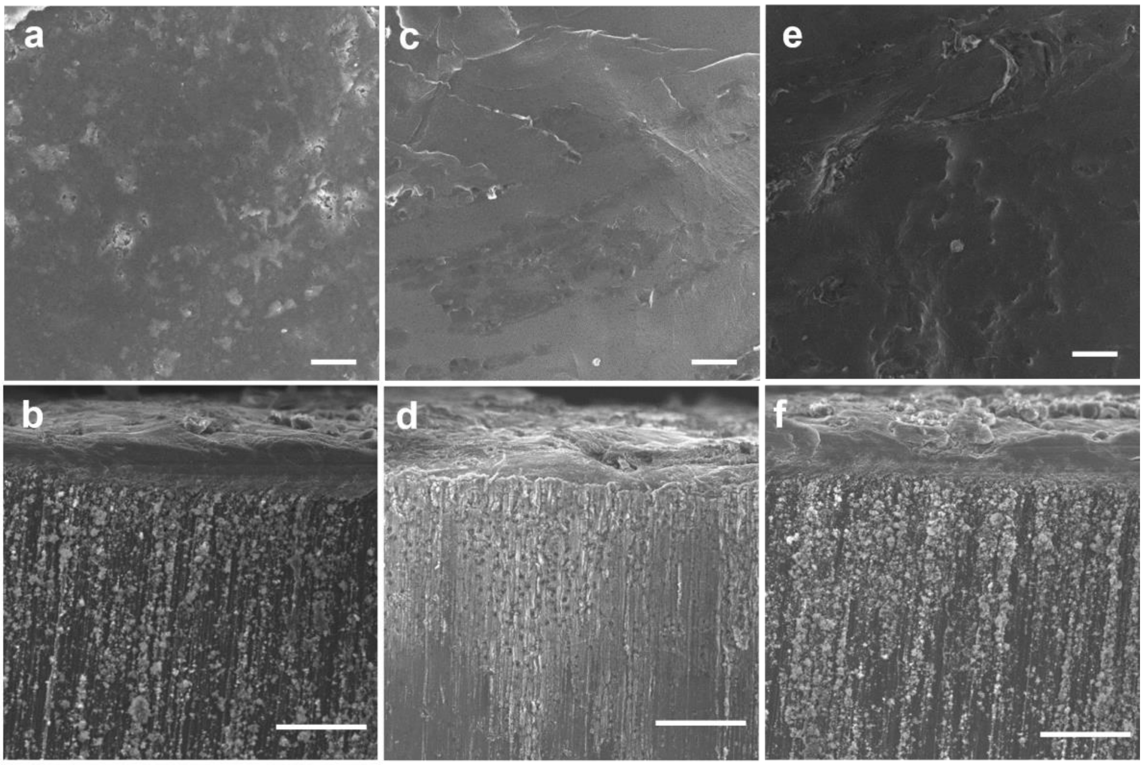

Supplementary Figure 23 | (a, c, e) Top-view and (b,d,f) cross-section SEM images of Li metal anodes assembled with GO-*g*-PAM@PP separators after (a, b) 1, (c, d) 10, and (e, f) 50 cycles. Scale bars, (a, c, e) 10 μm and (b, d, f) 50 μm.
